# Association Between Gamma-Glutamyl Transferase, Total Bilirubin and Systemic Lupus Erythematosus in Chinese Women

**DOI:** 10.3389/fimmu.2021.682400

**Published:** 2021-06-29

**Authors:** Wenran Zhang, Zhaoyang Tang, Yanjun Shi, Long Ji, Xueyu Chen, Yanru Chen, Xiaohui Wang, Meng Wang, Wei Wang, Dong Li

**Affiliations:** ^1^ Department of Epidemiology and Health Statistics, School of Public Health, Shandong First Medical University & Shandong Academy of Medical Sciences, Tai’an, China; ^2^ Department of Rheumatology and Immunology, Liaocheng People’s Hospital, Liao’cheng, China; ^3^ Clinical Research Center, The Second Affiliated Hospital of Shandong First Medical University, Tai’an, China

**Keywords:** systemic lupus erythematosus, total bilirubin, gamma-glutamyl transferase, female population, diagnostic marker, case-control study

## Abstract

**Background:**

Systemic lupus erythematosus (SLE) affects many organs and systems of the human organism, at present, its specific pathogenesis is not completely clear, but inflammation is considered to be an important factor involved in the pathogenesis and progression of SLE. Gamma-glutamyl transpeptidase (GGT) and total bilirubin (TBIL) have different effects on inflammation: GGT has pro-inflammatory effects, on the contrary, TBIL has anti-inflammatory effects. Study has found that GGT and TBIL play opposite roles in metabolic diseases. However, the roles of them in SLE are unknown. Meanwhile, the relationship between GGT and SLE also remains unexplored.

**Method:**

We recruited 341 SLE patients and 332 healthy individuals in Liaocheng People’s Hospital from August 2018 to May 2019. We diagnosed SLE using 2019 revised American College of Rheumatology (ACR) SLE criteria, and modeled the study outcomes using logistic regression to explore the respective relationship between GGT, TBIL and SLE. We also analyzed the interaction of GGT and TBIL in the progression of SLE.

**Results:**

We found that the levels of CRP, IL-6 and TNF-α in the aggravated group were significantly higher than those in the unaggravated group, the levels of C3 and C4 in the aggravated group were significantly lower than those in the unaggravated group. According to Spearman correlation analysis, GGT is proportional to CRP (r_s_=0.417) and IL-6 (r_s_=0.412), inversely proportional to C3 (r_s_=-0.177) and C4 (r_s_=0.-132). TBIL was inversely proportional to CRP (r_s_=-0.328) and TNF(r_s_=-0.360), and positively proportional to C3 (r_s_=0.174) and C4 (r_s_=0.172). In the fully adjusted model, compared to the lowest quartile, the highest quartile of GGT exhibited a positive association with the risk of SLE aggravation (OR=2.99, 95% CI: 1.42–6.31, *P*<0.001). At the same time, compared to the highest quartile, the quartile lowest of TBIL exhibited a positive association with the risk of SLE aggravation (OR=2.66, 95% CI: 1.27–5.59, *P*<0.001) in the fully adjusted model. Through interaction analysis, we found that women with high GGT levels had an increased risk of SLE aggravation when they had a low level of TBIL (OR=3.68, 95% CI: 1.51–9.01, for women with Q1 TBIL and Q4 GGT compared to women with Q2-Q4 TBIL and Q1-Q3 GGT, *P* for interaction <0.001), the combined AUC value (AUC_COMBINED_=0.711) of high GGT level and TBIL were higher than their respective values (AUC_GGT_=0.612, AUC_TBIL_=0.614).

**Conclusion:**

We found that the effects of GGT and TBIL in the progression of SLE are opposite. High GGT level might be a risk factor for SLE aggravation, as GGT levels increased, so did the risk of SLE aggravation. At the same time, we found that low TBIL level might be a risk factor for SLE aggravation. Moreover, high GGT level and low TBIL level had a subadditive effect on the increased risk of SLE aggravation.

## Introduction

Systemic lupus erythematosus (SLE) is a systemic autoimmune inflammatory disease that can affect many organs and systems of the human organism, such as the kidney, cardiovascular system, and respiratory system (1, 2). SLE is more common in females, the ratio of male to female is about 1:7 (3). SLE is a worldwide disease, the prevalence is about 100.3/100,000 in China, according to the study (4). In recent years, the prevalence of SLE has gradually increased, and the disease burden has gradually increased in China, SLE has become an important public health problem (5).

At present, the pathogenesis of SLE is not clear, but inflammation is considered to be an important factor involved in the pathogenesis and progression of SLE. Recent studies have found that the activation of many proinflammatory pathways caused SLE damage to multiple organs and tissues (6, 7). At the same time, various inflammatory markers represented by CRP play an important role in the evaluation of SLE disease progression (8).

Gamma-glutamyl transferase (GGT) is found mainly in the liver and plays an important role in maintaining intracellular glutathione concentration, so it is often used as a clinical indicator of potential liver or biliary tract diseases (9, 10). Recently, GGT has been reported to be associated with many chronic diseases, in these diseases, GGT has an obvious proinflammatory effect (11–14). Moreover, inflammation might be a possibly pathway leading to occurrence or aggravation of SLE. So, GGT levels might be associated with the risk of SLE’s occurrence or aggravation. However, few studies have examined the relationship between GGT levels and SLE. Therefore, the relationship between GGT and SLE is far from clear, especially among female individuals.

Bilirubin is a natural antioxidant present in biological fluids throughout the body and is among the most powerful endogenous antioxidants (15). Total bilirubin (TBIL) has been reported to be closely associated with immune diseases. The previous research indicates that the heme catabolism pathway plays a vital role in the immune system. Its products play an important role in cell protection and anti-inflammation in oxidative stress and inflammation (16). One population study showed that TBIL levels were lower in the case group than in the control group (17). We will investigate the role of low levels of TBIL in the female population to explore the true role of TBIL in SLE.

According to study, GGT and TBIL were found to have opposite roles in metabolic syndrome (18), but GGT and TBIL in SLE are unknown. We analyzed the interaction between GGT and TBIL to explore their influence on the pathogenesis and progression of SLE. At the same time, studies have found that both GGT and TBIL have sex differences in expression, and men are significantly higher than women (19, 20).

In summary, owing to the unknown relationship between GGT, TBIL and SLE, and the sex-specific expression of GGT and TBIL. We collected data on TBIL levels, GGT levels and general condition information from 673 female participants to explore the association between TBIL, GGT and SLE in Chinese women.

## Participants and Methods

### Study Population

In this case-control study, we included a total of 341 patients with SLE who were admitted to the Liaocheng People’s Hospital from August 2018 to May 2019. All patients were Chinese and met the 2019 revised American College of Rheumatology (ACR) SLE criteria (21). The participants who visited Liaocheng People’s Hospital for routine physical examination at the same given period were assigned to the control group. Based on the SLEDAI score and the involved organs, we divided the patients into two groups: the aggravated group (N=140) and the unaggravated group (N=201). The control group consisted of 332 individuals who were free from Immunologic diseases or Hemolysis diseases. Hepatobiliary and pancreatic diseases including cancer and viral hepatitis were excluded from this study, eventually, a total of 673 female participants were included in our study. Written informed consent was obtained from all participants.

### Assessment of Systemic Lupus Erythematosus

The current diagnostic criteria require a patient to present with 4 out of 11 symptoms/disorders, including cutaneous rashes, inflammation of the pleura or pericardium, inflammation of joints and muscles, renal and/or neurologic disorders, hematologic and immunologic disorders, and most significantly, autoantibodies specifically targeting nuclear antigens (such as double-stranded DNA, small nuclear riboproteins, chromatin, and histone proteins), or to a lesser extent, cytoplasmic antigens (21). We use the SLEDAI score and involved organs to judge the activity and progression of SLE.

### Assessment of Gamma-Glutamyl Transferase (GGT) and Total Bilirubin (TBIL)

Blood samples were collected by venipuncture from the large antecubital veins in the morning after overnight fasting. All blood samples were stored in vacuum tubes containing ethylenediaminetetraacetic acid (EDTA). GGT and TBIL levels were determined using an autoanalyzer (Hitachi 747; Hitachi, Tokyo, Japan) with the kinetic and uricase-peroxidase methods, respectively, in the laboratories of Liaocheng People’s Hospital.

### Assessment of Covariates

Well-trained interviewers administered a standardized questionnaire to collect participants’ information. Demographic variables, including age, sex and menstrual history were collected through the questionnaire. The menstrual history was asked to see if they had gone through menopause and their age at menopause. BMI was defined based on the measured height and weight and calculated as weight (kg)/height (m2).

The diagnostic criterion of hypertension was as follows: systolic blood pressure ≥140 mmHg and/or diastolic blood pressure ≥90 mmHg. Diabetes mellitus was defined as the fasting blood glucose level ≥ 7.0 mmol/L (126 mg/dL). Dyslipidemia was defined as low-density lipoprotein (LDL) ≥ 3.37 mmol/L, high-density lipoprotein (HDL) < 1.04 mmol/L, total cholesterol (TC) ≥ 5.18 mmol/L, or serum levels of triglyceride (TG) ≥ 1.7 mmol/L.

The levels of C-reactive protein (CRP), Tumor Necrosis Factor-alpha (TNF-α), interleukin-2(IL-2) interleukin-4(IL-4), interleukin-6 (IL-6), interleukin-10 (IL-10), IgG, IgA, and IgM were determined by the double antibody sandwich ELISA method. Immunoturbidimetry was performed to measure the concentration of C3 and C4 using an Immunoturbidimetry kit. Antinuclear antibody was assessed by indirect fluorescence, positive of anti-SM, positive of anti-SSA, positive of anti-SSB, positive of anti-dsDNA and positive of anti-RNP.

### Statistical Analysi*s*

Kolmogorov-Smirnov tests were used to test the normality of the continuous variables. Continuous variables are presented as the mean with standard deviation and were compared using the independent-sample t test; meanwhile, variables with a skewed distribution are presented as the median [interquartile range (22)]; Categorical variables were compared using the chi‐square test. Spearman correlation was used to explore the separate correlation between GGT, TBIL and inflammatory and immunological indexes. Both TBIL and GGT were categorized into quartiles using the 25th, 50th, and 75th percentiles in different study groups. Logistic regression was used to evaluate GGT, TBIL and SLE’s association by calculating the odds ratio (OR) or adjusted OR with 95% confidence interval (CI). To examine effect modification by GGT and TBIL, we used a post‐estimation Wald test in a multivariable‐adjusted logistic model to obtain an omnibus P value for the interaction of GGT and IBIL and the interactive effect of GGT and TBIL in progression of SLE. ROC curve was based on the results of logistic regression analysis to evaluate the value of combined diagnosis of GGT and TBIL in the progression of SLE.

All statistical tests were two‐sided, and the significance level was set as *P* < 0.05. The statistical analyses were performed using SAS software, version 9.4 (SAS Institute Inc., Cary, NC, USA).

## Results

### Baseline Characteristics of the Study Participants

A total of 673 participants from Liaocheng People’s Hospital eventually enrolled in our study. The characteristics of the participants are summarized in **Table 1**. Significant differences were found among the groups for Age, TBIL, GGT, ALT, SUA, Mild-moderate drinking, Hypertension, GLU, TC, HDL and LDL (P<0.001). The level of GGT in case group was significantly higher than that in control group, on the contrary, the level of TBIL in control group was significantly lower than that in control group.

**Table 1:** Baseline characteristics.

Characteristic	SLE			Control	P
	Total (N=341)	Unaggravated Group (N=201)	Aggravated Group (N=140)	(N=332)	
Age (mean ± SD)	40.46 ± 12.92	40.71 ± 0.77	39.09 ± 1.66	49.10 ± 12.07	<0.001
GGT (U/L)	20.00 (14.00,31.00)	17.00 (13.00,27.00)	23.00 (13.00,42.00)	16.00 (12.25,22.75)	<0.001
TBIL (mg/L)	10.90 (8.30,13.80)	11.50 (8.52,14.97)	10.35 (8.10,12.70)	13.10 (10.50,16.70)	<0.001
ALT(U/L)	23.00 (17.00,30.00)	15.00 (12.00,21.00)	22.00 (17.00,29.75)	27.00 (12.00,32.00)	<0.001
AST(U/L)	22.00 (20.00,27.00)	23.00 (20.00,27.00)	21.00 (17.25,26.75)	23.00 (16.00,28.00)	0.652
SUA (umol/L)	257.00 (194.50,306.00)	269.00 (199.00,315.00)	233.00 (188.25,281.75)	295.00 (237.25,352.00)	<0.001
Menopause (n, %)	104 (30.50)	85 (42.28)	19 (13.57)	81 (24.40)	0.076
Current smoker (n, %)	8 (2.34)	4 (0.19)	4 (2.85)	3 (0.09)	0.141
Mild-moderate drinking (n, %)	57 (16.71)	43 (21.39)	14 (10.00)	5 (1.51)	<0.001
BMI (mean ± SD)	23.92 ± 3.15	23.98 ± 0.19	23.62 ± 0.35	23.65 ± 2.75	0.244
Hypertension (n, %)	33 (9.68)	26 (12.93)	7 (5.00)	12 (3.61)	<0.001
TC (mmol/L)	4.56 (3.78,5.63)	4.58 (3.80,5.70)	4.52 (3.57,5.30)	5.08 (4.40,5.74)	<0.001
TG (mmol/L)	1.27 (0.91,1.81)	1.31 (0.96,1.79)	1.21 (0.81,1.90)	1.19 (0.80,1.81)	0.225
HDL (mmol/L)	1.24 (1.04,1.56)	1.23 (1.04,1.55)	1.27 (1.01,1.57)	1.48 (1.27,1.72)	<0.001
LDL (mmol/L)	2.81 (1.71,3.91)	2.64 (2.02,3.69)	2.54 (1.78,2.97)	2.04 (1.42,2.66)	<0.001
GLU (mmol/L)	5.64 (3.83,7.45)	5.18 (4.75,5.90)	5.23 (4.79,6.02)	5.77 (4.47,7.07)	<0.001

Unaggravated Group: SLEDAI Score < 10 and involved the skin, mucous membranes or joints.

Aggravated Group: SLEDAI Score > 10 and involved the kidneys, lungs, or cardiovascular system.

### Inflammatory and Immunological Index in SLE Patients

We included patients in the unaggravated group with a SLEDAI score of less than 10 and involved only small organs such as skin, mucous membranes, or joints. In the meantime, patients were included in the aggravated group with a SLEDAI score of more than 10 and involved mainly organs such as kidneys, lungs, or cardiovascular system. We found that the levels of CRP, IL-6 and TNF-α in the aggravated group were significantly higher than those in the unaggravated group, the levels of C3 and C4 in the aggravated group were significantly lower than those in the unaggravated group. According to Spearman correlation analysis, GGT is proportional to CRP (r_s_=0.417) and IL-6 (r_s_=0.412), inversely proportional to C3 (r_s_=-0.177) and C4 (r_s_=0.-132). TBIL was inversely proportional to CRP (r_s_=-0.328) and TNF(r_s_=-0.360), and positively proportional to C3 (r_s_=0.174) and C4 (r_s_=0.172) (**Table 2**).

**Table 2:** Inflammatory and Immunological index in SLE patients.

Characteristic		SLE				GGT		TBIL	
		Total	Unaggravated Group	Aggravated Group	*P*	rs	*P*	rs	*P*
Inflammatory index	CRP (mg/L)	8.64 (3.41,19.83)	7.75 (3.22,15.23)	10.21 (3.68,41.27)	0.034*	0.417	<0.001*	-0.328	<0.001*
	IL-2 (pg/mL)	0.30 (0.10,0.63)	0.22 (0.10,0.50)	0.33 (0.10,0.66)	0.063	0.022	0.686	0.075	0.169
	IL-4 (pg/mL)	0.20 (0.10,0.50)	0.20 (0.10,0.52)	0.25 (0.10,0.47)	0.231	0.039	0.47	0.039	0.478
	IL-6 (pg/mL)	23.42 (11.39,63.09)	19.1 (10.08,49.08)	32.97 (15.95,111.52)	<0.001*	0.412	<0.001*	0.015	0.779
	IL-10 (pg/mL)	2.61 (1.08,5.52)	2.61 (1.21,5.36)	2.76 (0.99,5.84)	0.879	0.015	0.786	-0.054	0.316
	TNF-α (pg/mL)	15.79 (11.00,32.76)	13.8 (10.17,24.96)	20.14 (12.25,41.72)	<0.001*	0.001	0.98	-0.36	<0.001*
	IgG (mg/L)	15.30 (12.00,19.35)	15.30 (12.30,19.75)	15.20 (11.80,19.22)	0.459	-0.075	0.166	0.01	0.848
	IgA (mg/L)	2.71 (1.88,3.58)	2.74 (1.95,3.58)	2.69 (1.74,3.58)	0.678	-0.068	0.211	0.03	0.586
	IgM (mg/L)	0.90 (0.60,1.31)	0.93 (0.61,1.31)	0.85 (0.58,1.31)	0.684	-0.041	0.45	0.013	0.807
Immunological Index	C3 (g/L)	0.66 (0.41,0.87)	0.70 (0.53,0.93)	0.53 (0.36,0.74)	<0.001*	-0.177	<0.001*	0.174	0.001*
	C4 (g/L)	0.13 (0.08,0.21)	0.14 (0.10,0.23)	0.12 (0.08,0.16)	0.001*	-0.132	0.015*	0.172	0.001*
	Positive of anti-SM (n, %)	145 (42.52)	81 (40.29)	64 (45.71)	0.32	-0.009	0.864	-0.001	0.982
	Positive of anti-SSA (n, %)	212 (62.17)	115 (57.21)	97 (69.28)	0.024*	-0.011	0.87	0.02	0.716
	Positive of anti-SSB (n, %)	61 (17.88)	30 (14.92)	31 (22.14)	0.087	-0.074	0.174	0.018	0.739
	Positive of anti-dsDNA (n, %)	174 (51.02)	109 (54.23)	65 (46.42)	0.156	-0.127	0.019*	0.011	0.839
	Positive of anti-RNP (n, %)	103 (30.21)	59 (29.35)	44 (31.42)	0.681	0.019	0.732	0.022	0.69
SLEDAI		8 (3,12)	5 (3,8)	12 (10,15)	<0.001*	0.188	<0.001*	-0.121	0.026*

*P < 0.05.

### Effect of GGT and TBIL in Occurrence and Progression of SLE

**Figure 1:**
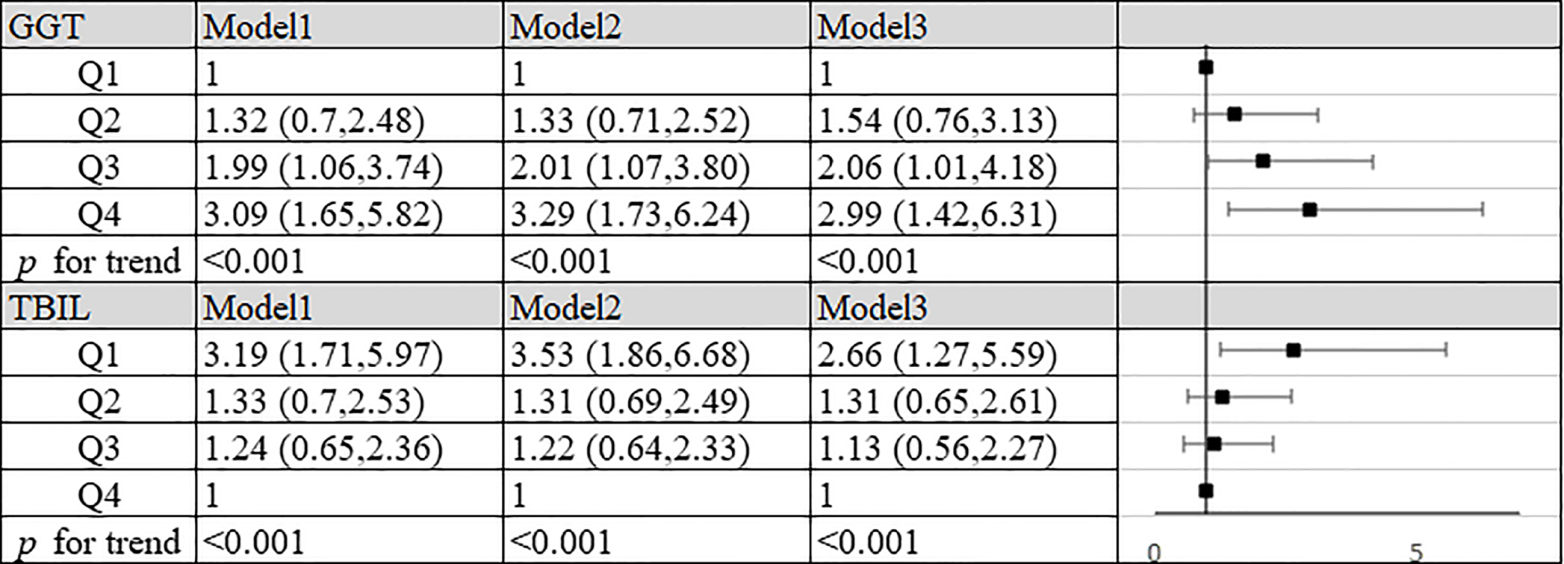
Association of TBIL, GGT and aggravation of SLE in patients. Model 1: unadjusted. Model 2: adjusted for age, BMI, smoking status, alcohol consumption and estrogen level. Model 3: adjusted for age, BMI, smoking status, alcohol consumption, estrogen level, ALT, AST, SUA, CRP, IL-2, IL-4, IL-6, IL-10, TNF-α, hypertension, hyperlipidemia and diabetes mellitus. GGT: Q1, quartile 1 (n=89): ≤14 U/L; Q2, quartile 2 (n=88): 15-20 U/L; Q3, quartile 3 (n=82): 21-31 U/L; Q4, quartile 4 (n=82): >31 U/L. TBIL: Q1, quartile 1 (n=90): ≤8.3 mg/L; Q2, quartile 2 (n=83): 8.4-10.9 mg/L; Q3, quartile 3 (n=84): 11.0-13.8 mg/L; Q4, quartile 4 (n=84): >13.8 mg/L.

In order to explore the role of GGT and TBIL in the occurrence of SLE, we combined the unaggravated group with the control group, and the aggravated group with the control group. By Logistic regression analysis, we found that most of the OR values obtained were not statistically significant or the 95% confidence interval of ORs were very close to the void line, suggesting that GGT and TBIL may be of low value in the occurrence of SLE, pleases see **Supplementary Figures 1** and **2** for details. In further research, we focused on exploring the role of GGT and TBIL in the progression of SLE. We combined the unaggravated group with the aggravated group, by Logistic regression analysis, we found that compared to the lowest quartile, the highest quartile of GGT exhibited a positive association with progression of SLE risk (OR=2.99, 95% CI: 1.42–6.31, *P*<0.001) in the fully adjusted model. In all three models, the values of *P* for trend across quartiles were less than 0.001 (**Figure 1**). In the meantime, we found that compared to the highest quartile, the quartile lowest of TBIL exhibited a positive association with the progression of SLE risk (OR=2.66, 95% CI: 1.27–5.59, *P*<0.001) in the fully adjusted model. In all three models, the values of *P* for trend across quartiles were less than 0.001 (**Figure 1**).

### Interactive Effect Between GGT and TBIL in the Progression of SLE

**Figure 2:**
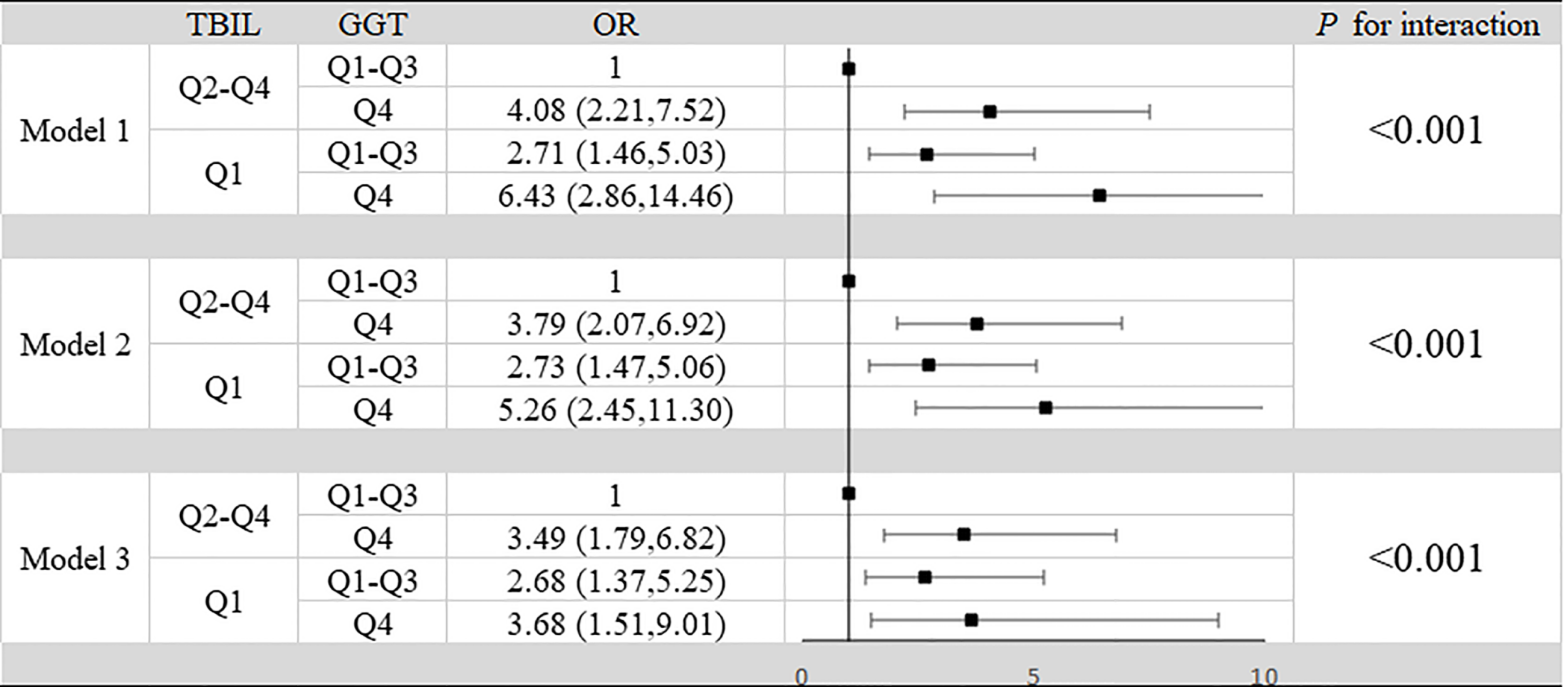
Interaction between TBIL and GGT in aggravation of SLE in patients. Model 1: unadjusted. Model 2: adjusted for age, BMI, smoking status, alcohol consumption and estrogen level. Model 3: adjusted for age, BMI, smoking status, alcohol consumption, estrogen level, ALT, AST, SUA, CRP, IL-2, IL-4, IL-6, IL-10, TNF-α, hypertension, hyperlipidemia and diabetes mellitus.

**Figure 3:**
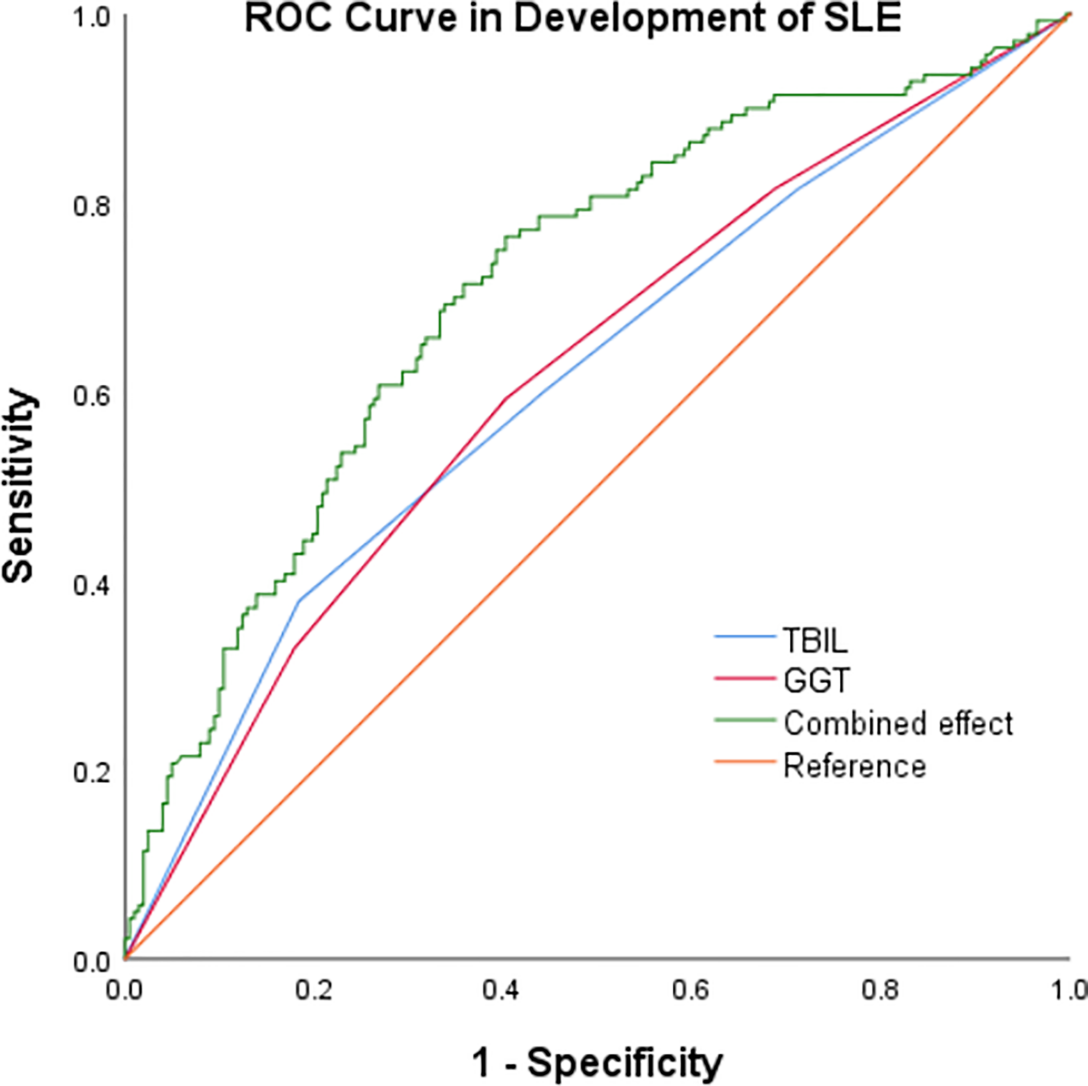
ROC curve of TBIL, GGT and the combined effect in development of SLE.

In our study, high GGT level and low TBIL level increased the risk of progression of SLE respectively. At the same time we summarized the analysis results on the effects of the interactions between TBIL and GGT on the risk of SLE aggravation in **Figure 2**. According to interaction analysis, we found that women with high GGT levels had an increased risk of SLE aggravation when they had a low level of TBIL (OR=3.68, 95% CI: 1.51–9.01, for women with Q1 TBIL and Q4 GGT compared to women with Q2-Q4 TBIL and Q1-Q3 GGT, *P* for interaction <0.001). Considering women with low levels of TBIL, when they had high GGT levels, we obtained higher OR values than that we did for women with low TBIL levels alone (OR=2.66, 95% CI: 1.27–5.59, *P*<0.001) or women with high GGT levels alone (OR=2.99, 95% CI: 1.42–6.31, *P*<0.001). So GGT and TBIL had a subadditive effect on the increased risk of SLE (**Figure 1**). Logistic regression was used to construct the ROC curve, we found that the combined AUC value (AUC_COMBINED_=0.711) of high GGT level and TBIL were higher than their respective values (AUC_GGT_=0.612, AUC_TBIL_=0.614) (**Figure 3**).

## Discussion

Our study found that the roles of GGT and TBIL were reversed in SLE aggravation, which is consistent with previous studies on metabolic syndrome (18). With the increase of GGT and decrease of TBIL, the risk of SLE aggravation increased.

Precious study showed that SLE affected women of childbearing age are about nine times more frequent than men (23), which indicated that women are more likely to suffer from SLE. Simultaneously, for GGT and TBIL, the female population is also unique in its expression and activity. An ovariectomized mouse model found that GGT activity might be related to estrogen (19). A large population study also found that men had significantly higher levels of TBIL than women (20). Therefore, it is important to explore the relationship between GGT, TBIL and SLE in the female population.

Systemic lupus erythematosus (SLE) is a kind of systemic autoimmune inflammatory disease. The aggravation of the disease is often accompanied by inflammation of various organs. Previous studies have found that CRP may be an excellent indicator of inflammatory progression in SLE patients, and our study also found a significant increase in CRP levels in patients with SLE aggravation (8, 24). IL-6 and TNF-α were also thought to be elevated in SLE aggravation, which is also consistent with our findings (25). Moreover, previous studies have shown a protective effect of complement on SLE, which is consistent with our findings (26, 27). According to the study, we found that the proinflammatory effect of GGT may be through the promotion of IL-6 levels, which leads to CRP levels rise, consumption of more complement, making the increase of GGT become a risk factor for SLE aggravation (28, 29). At the same time, we also found that the anti-inflammatory effect of TBIL may be reflected by inhibiting TNF-α levels, which leads to the decrease of CRP level and decrease of complement consumption, making the decrease of TBIL a risk factor for SLE aggravation (30–33).

Our study found that elevated GGT may increase the risk of SLE aggravation, which may be due to the strong pro-inflammatory effects of GGT. Although no studies have been done on GGT and SLE’s relationship, GGT has been involved in various chronic diseases in recent years, such as arteriosclerosis, heart disease, and diabetes. In these diseases, GGT plays a key role in promoting inflammation (13, 34). It is reasonable to assume that GGT is also involved in the pathogenesis of SLE aggravation.

Our study found that TBIL levels were lower in the case group than in the control group, consistent with previous findings (17). There are two possible explanations: One is the anti-inflammatory effect of bilirubin. It has been suggested that bilirubin has potent anti-inflammatory activities in brain tissue. This effect has been well demonstrated in experimental autoimmune encephalomyelitis (32). Thus, mildly elevated levels of bilirubin may confer protection against the deleterious effects of inflammations, also, a lack of TBIL would have the opposite effect. The other explanation is the powerful immunoregulatory effect of bilirubin. Complement plays an important role in cleaning away bacteria, releasing harmful substances, and increasing disease resistance (30). Various autoantibodies react with antibodies to form antigen-antibody immune complexes in the serum of patients with SLE. Large amounts of immune complexes exist in the blood and tissues, which activate and bind and then deplete it (35, 36). This reaction can be activated *via* the classical pathway and is initiated by binding the C1 component to antigen-bound antibodies, known as immunocomplexes. Mildly elevated bilirubin levels have a negative effect in the SLE by interfering with the interaction between C1 and immunoglobulin, this interference does not occur when TBIL is absent (37).

Above all, GGT has pro-inflammatory effects, elevated GGT may increase the risk of SLE aggravation. While TBIL has anti-inflammatory effects, decreased TBIL may also increase the risk of SLE aggravation. GGT and TBIL have opposite effect in SLE aggravation may be reflected in the roles of CRP expression.

## Conclusion

In our case-control study, we found that the roles of GGT and TBIL in the progression of SLE are opposite. High GGT level might be a risk factor for SLE aggravation in the female population, and as GGT levels increased, so did the risk of SLE aggravation. In the meanwhile, we also found that women with low TBIL level were at increased risk of SLE aggravation. Moreover, high GGT level and low TBIL level had a subadditive effect on the increased risk of SLE aggravation. This suggests that the patients of SLE is at higher risk to make SLE more aggravated when GGT levels are elevated and TBIL levels are reduced in the female population. The combined diagnosis of GGT and TBIL is valuable for the progression of SLE.

## Data Availability Statement

The original contributions presented in the study are included in the article/**Supplementary Material**, further inquiries can be directed to the corresponding authors.

## Ethics Statement

The studies involving human participants were reviewed and approved by Ethics Committee of Shandong First Medical University and Liaocheng People’s Hospital. The patients/participants provided their written informed consent to participate in this study.

## Author Contributions

All authors contributed to the article and approved the submitted version. WZ and ZT contributed equally to this work.

## Funding

This work was supported by the National Natural Science Foundation of China (81773527).

## Conflict of Interest

The authors declare that the research was conducted in the absence of any commercial or financial relationships that could be construed as a potential conflict of interest.

## Supplementary Material

Supplementary Figure 1 Association of GGT, TBIL and SLE in inactive and control group. Model 1: unadjusted. Model 2: adjusted for age, BMI, smoking status, alcohol consumption and estrogen level. Model 3: adjusted for age, BMI, smoking status, alcohol consumption, estrogen level, ALT, AST, SUA, hypertension, hyperlipidemia and diabetes mellitus. GGT: Q1, quartile 1 (n=160): ≤13 U/L; Q2, quartile 2 (n=123): 14-17 U/L; Q3, quartile 3 (n=125): 18-25 U/L; Q4, quartile 4 (n=125): >25 U/L. TBIL: Q1, quartile 1 (n=140): ≤9.9 mg/L; Q2, quartile 2 (n=130):10.0-12.6 mg/L; Q3, quartile 3 (n=133): 12.7-16.4 mg/L; Q4, quartile 4 (n=131): >16.4 mg/L.

Supplementary Figure 2 Association of GGT, TBIL and SLE in active and control group. Model 1: unadjusted. Model 2: adjusted for age, BMI, smoking status, alcohol consumption and estrogen level. Model 3: adjusted for age, BMI, smoking status, alcohol consumption, estrogen level, ALT, AST, SUA, hypertension, hyperlipidemia and diabetes mellitus. GGT: Q1, quartile 1 (n=126): ≤13 U/L; Q2, quartile 2 (n=112): 14-18 U/L; Q3, quartile 3 (n=122): 19-28 U/L; Q4, quartile 4 (n=113): >28 U/L. TBIL: Q1, quartile 1 (n=115): ≤9.6 mg/L; Q2, quartile 2 (n=118): 9.7-12.3 mg/L; Q3, quartile 3 (n=121): 12.4-15.7 mg/L; Q4, quartile 4 (n=118): >15.7 mg/L.
