# Supplementary figures and images for: Association Between Gamma-Glutamyl Transferase, Total Bilirubin and Systemic Lupus Erythematosus in Chinese Women
Source: Front Immunol. 2021 Jun 29;12:682400. doi: 10.3389/fimmu.2021.682400 (PMC8277571; doi:10.3389/fimmu.2021.682400)

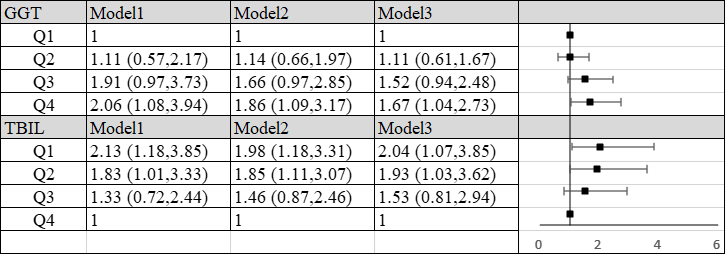

Supplement: Supplementary Figure 1 — Association of GGT, TBIL and SLE in inactive and control group. Model 1: unadjusted. Model 2: adjusted for age, BMI, smoking status, alcohol consumption and estrogen level. Model 3: adjusted for age, BMI, smoking status, alcohol consumption, estrogen level, ALT, AST, SUA, hypertension, hyperlipidemia and diabetes mellitus. GGT: Q1, quartile 1 (n=160): ≤13 U/L; Q2, quartile 2 (n=123): 14-17 U/L; Q3, quartile 3 (n=125): 18-25 U/L; Q4, quartile 4 (n=125): >25 U/L. TBIL: Q1, quartile 1 (n=140): ≤9.9 mg/L; Q2, quartile 2 (n=130):10.0-12.6 mg/L; Q3, quartile 3 (n=133): 12.7-16.4 mg/L; Q4, quartile 4 (n=131): >16.4 mg/L. [file Image_1.tif]

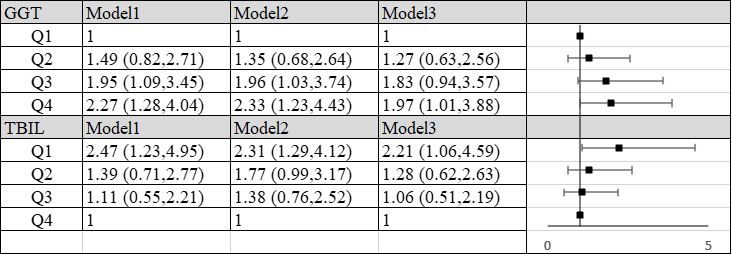

Supplement: Supplementary Figure 2 — Association of GGT, TBIL and SLE in active and control group. Model 1: unadjusted. Model 2: adjusted for age, BMI, smoking status, alcohol consumption and estrogen level. Model 3: adjusted for age, BMI, smoking status, alcohol consumption, estrogen level, ALT, AST, SUA, hypertension, hyperlipidemia and diabetes mellitus. GGT: Q1, quartile 1 (n=126): ≤13 U/L; Q2, quartile 2 (n=112): 14-18 U/L; Q3, quartile 3 (n=122): 19-28 U/L; Q4, quartile 4 (n=113): >28 U/L. TBIL: Q1, quartile 1 (n=115): ≤9.6 mg/L; Q2, quartile 2 (n=118): 9.7-12.3 mg/L; Q3, quartile 3 (n=121): 12.4-15.7 mg/L; Q4, quartile 4 (n=118): >15.7 mg/L. [file Image_2.tif]
